# Intracranial pressure directly predicts headache morbidity in idiopathic intracranial hypertension

**DOI:** 10.1186/s10194-021-01321-8

**Published:** 2021-10-07

**Authors:** S. P. Mollan, B. R. Wakerley, Z. Alimajstorovic, J. Mitchell, R. Ottridge, A. Yiangou, M. Thaller, A. Gupta, O. Grech, G. Lavery, K. Brock, A. J. Sinclair

**Affiliations:** 1 Birmingham Neuro-Ophthalmology Unit, University Hospitals Birmingham, Birmingham, UK; 2 University of Birmingham, Metabolic Neurology, Institute of Metabolism and Systems Research, Birmingham, UK; 3 Department of Neurology, University Hospitals Birmingham, Birmingham, UK; 4 University of Birmingham College of Medical and Dental Sciences, Birmingham, UK; 5 Birmingham Clinical Trials Unit, University of Birmingham, Birmingham, UK

**Keywords:** Idiopathic intracranial hypertension, Migraine, Intracranial pressure, Allodynia, Calcitonin gene related peptide

## Abstract

**Objective:**

Headache is the predominant disabler in idiopathic intracranial hypertension (IIH). The aim was to characterise headache and investigate the association with intracranial pressure.

**Methods:**

IIH:WT was a randomised controlled parallel group multicentre trial in the United Kingdom investigating weight management methods in IIH. Participants with active IIH (evidenced by papilloedema) and a body mass index (BMI) ≥35 kg/m^2^ were recruited. At baseline, 12 months and 24 months headache characteristics and quality of life outcome measures were collected and lumbar puncture measurements were performed.

**Results:**

Sixty-six women with active IIH were included with a mean age of 32.0 years (SD ± 7.8), and mean body mass index of 43.9 ± 7.0 kg/m^2^. The headache phenotype was migraine-like in 90%. Headache severity correlated with ICP at baseline (*r =* 0.285; *p* = 0.024); change in headache severity and monthly headache days correlated with change in ICP at 12 months (*r =* 0.454, *p* = 0.001 and *r =* 0.419, *p* = 0.002 respectively). Cutaneous allodynia was significantly correlated with ICP at 12 months. (*r =* 0.479, *p* < 0.001). Boot strap analysis noted a positive association between ICP at 12 and 24 months and enabled prediction of both change in headache severity and monthly headache days. ICP was associated with significant improvements in quality of life (SF-36).

**Conclusions:**

We demonstrate a positive relationship between ICP and headache and cutaneous allodynia, which has not been previously reported in IIH. Those with the greatest reduction in ICP over 12 months had the greatest reduction in headache frequency and severity; this was associated with improvement of quality of life measures.

**Trial registration:**

This work provides Class IIa evidence of the association of raised intracranial pressure and headache. ClinicalTrials.gov number, NCT02124486.

**Supplementary Information:**

The online version contains supplementary material available at 10.1186/s10194-021-01321-8.

## Introduction

Idiopathic intracranial hypertension (IIH) is a rare disease that is increasingly recognised [1, 2]. It is characterized by raised intracranial pressure (ICP) in the absence of a structural cause on brain imaging [3, 4]. Although the exact cause of IIH remains unknown, disease development is associated with obesity and there is increasing evidence to suggest adipose dysfunction [5]. Headache is the predominant symptom and is prioritized highly by patients and physicians [6]. The headache in IIH remains under-characterized [7] and is associated with significant morbidity and reduced quality of life [8].

The International Classification of Headache Disorders (ICHD-3 beta) attributes headache in IIH to be associated with raised intracranial pressure and acknowledges that it often mimics primary headache disorders such as migraine [9]. While the International Headache Society stipulates the importance on relief of headache following removal of cerebrospinal fluid (CSF), evidence suggests this relief is far from universal [10] and can occur due to co-occurring headache disorders such as migraine [11]. Rarely some patients with evidence of raised ICP do not ever develop headache [12]. The pathogenesis of IIH headache remains unknown, as do the risk factors that propagate persistent headache. Calcitonin gene-related peptide (CGRP) monoclonal antibodies have been noted in a prospective study to significantly improve headache in patients with IIH and persistent post-IIH headache (resolved papilloedema), suggesting CGRP may modulate headache pain [13, 14].

The relationship between development of headache and raised ICP in IIH is poorly understood. A previous trial in 165 patients with newly diagnosed IIH [15] did not identify an association between intracranial pressure and headache. However, a question remains as to whether the role of ICP in driving ongoing headache in patients with chronic headache.

The aim of this analysis was to describe the headache characteristics in those with active IIH recruited to the multicentre IIH Weight Trial (IIH:WT), and to explore the relationship of headache features to ICP.

## Methods

### Study procedures

Between July 25, 2014 and May 25, 2017, 66 female patients were recruited to the multicentre randomised controlled trial IIH:WT, comparing the efficacy of a bariatric surgery pathway versus the dietary intervention Weight Watchers™. All participants had active IIH with papilloedema and lumbar puncture opening pressures ≥25 cm cerebrospinal fluid, in accordance with agreed criteria for diagnosis of IIH [1]. The protocol and eligibility criteria have been previously published [16].

At baseline a detailed clinical, medication and standardized headache history was taken (including the location, character, associated symptoms, timing and exacerbating / relieving factors) by a physician with specialist training in headache phenotyping. Headache preventatives were permitted during the study, but any changes were recorded. As part of the trial anthropometric data including weight and height were recorded, and a lumbar puncture was performed. Headaches were characterized using ICHD-3beta criteria for primary and secondary headache disorders [9].

At baseline, 12 and 24 months all IIH patients were required to return a headache diary which included details of headache severity; headache duration; headache frequency (monthly headache days); and analgesic use (days per month). The headache severity was scored using a numerical rating scale (NRS) ranging from 0 (no pain) to 10 (the most severe pain level experienced by the subject). The NRS is favoured by patients and widely used in migraine trials [17].

Cutaneous allodynia symptoms were assessed during a headache using the patient-completed Allodynia Symptom Checklist-12 (ASC-12) in the week prior to their baseline and 12 month visit. Score ranges from 0 = no symptoms to 24 = severe symptoms; 0–2 = no allodynia, 3–5 = mild allodynia, 6–8 = moderate allodynia, 9 or more = severe allodynia (supplemental methods). In addition, pressure allodynia was assessed in patients at baseline and at 12 months using three different weights of von Frey hairs (F1, 0.32 g; F2, 8.30 g; and F3, 24 g) over V1–3 and C2–3 dermatomes bilaterally [18]. Patients rated pressure allodynia using a 100 mm visual analogue scale.

Outcome measures included the headache impact test-6 disability questionnaire (HIT-6); where little or no impact = HIT-6 score ≤ 49; some impact = HIT-6 score 50–55; substantial impact = HIT-6 score 56–59; severe impact = HIT-6 score ≥ 60. Health-related quality of life was assessed using the Rand patient-reported 36-Item Short Form Health Survey (SF-36). The eight sections of the SF-36 yielded two summary scores (physical component summary, PCS; and mental component summary, MCS).

### Statistical analysis

Descriptive statistics were used to compare demographic characteristics. Analysis by trial arm was not part of the aims of the study. Statistical analysis was performed using GraphPad, version 8.3 (GraphPad Software, La Jolla, California, USA). Mean and standard deviations are provided for normally distributed variables, and median and range provided for non-normally distributed variables. Pearson’s correlation coefficient was computed where the variables were normally distributed and all assumptions were met, with Spearman’s rank correlation used in other cases. Values were deemed statistically significant at *p* < 0.05. Missing data, due to any absence or choice, were excluded from the analysis and not imputed.

Hierarchical regression models were generated, with data for all patients analysed in one model. Models contained population-level terms (i.e. terms that apply to each experimental unit) to reflect: 1) the mean baseline value (i.e. the intercept); 2) the mean change from baseline associated with each assessment time (i.e. time as a factor variable); 3) the extra mean change from baseline associated with each assessment time in the experimental arm (i.e. the interaction of treatment allocation and time as a factor variable). Additionally, hierarchical regression models contained random effects (i.e. terms that are specific to each experimental unit) to reflect the random deviations from the population-level mean value at baseline (i.e. random intercepts). For ICP, the random intercepts were estimated for each patient, with each of these parameters assumed to be exchangeable draws from a normal distribution.

To assess the relationship between ICP and headache, we have bootstrap resampling of the observed outcomes to generate alternative pairs of treatment effects. Alternative datasets were generated by resampling patients with replacement from the original treatment allocations to which they were randomised. The hierarchical regression model described above was fitted to each resampled dataset, producing a pair of treatment effects for the surrogate and clinical outcomes from a notional randomisation of patients. This process was repeated 1000 times. The resampled datasets within in each arm in each trial were the same size as the original datasets.

### Standard protocol approvals, registration, and patient consent

The trial was approved by The National Research Ethics Committee West Midlands – The Black Country, on 28 February 2014 (14/WM/0011). All participants gave written consent after receiving detailed written information. The trial was registered, clinicaltrials.gov identifier: NCT02124486.

### Data availability statement

Anonymised individual participant data will be made available along with the trial protocol and statistical analysis plan. Proposals should be made to the corresponding author and will be reviewed by the Birmingham Clinical Trials Unit Data Sharing Committee in discussion with the Chief Investigator. A formal Data Sharing Agreement may be required between respective organisations once release of the data is approved and before data can be released.

### Classification of evidence

This work provides Class IIa evidence that headache is associated with intracranial pressure.

## Results

### Patient characteristics

Sixty-six persons (100% women) with active IIH were included in the analysis (Table 1). Mean age at inclusion was 32.0 years (SD ±7.8, range 20–53 years), and the mean body mass index was 43.9 ± 7.0 kg/m^2^ ranging from 35.3 to 63.3 kg/m^2^. The median IIH disease duration was 1.1 years (IQR 0.5–2.6) and ranging from 0.1 to 20.0 years. Forty-five participants (68%) reported a previous history of migraine, of which 24 (53%) reported onset in childhood (age < 18 years).

**Table 1:** Characteristics of the study cohort at baseline

	Total (***n*** = 66)
Age in years, mean (SD)	32 (7.8)
Ethnicity, number (%)	
White	55 (83)
Mixed	5 (8)
Asian	1 (1)
Black	5 (8)
Duration of IIH diagnosis, median (IQR)	1.1 (0.5–2.6)
Number on acetazolamide (%)	19 (29)
Number on topiramate (%)	6 (9)
Lumbar puncture opening pressure, cmCSF, mean (SD)	35.5 (7.0)
Weight, Kg (SD)	118.5 (21.1)
Body Mass Index (weight (kg)/ height (m^2^), mean (SD)	43.9 (7.0)
Smoking status - smoker	*n* = 27
Smoking intensity of 10 or more cigarettes per day, n (%)	18 (67)
Family history of primary headache disorder, %	19 (7/37)
Previous history of migraine, n (%)	45 (68)
Duration of migraine, n (%)	*n* = 45
Less than 1 year	5 (11)
1–5 years	7 (16)
5–10 years	5 (11)
10–20 years	3 (7)
More than 20 years	1 (2)
Since childhood (age < 18 years)	24 (53)
Headache preventative medication use, n (%)	*n* = 18
Beta-blocker	1 (6)
Tricyclic antidepressant	7 (39)
Anticonvulsant	8 (44)
Other	2 (11)

### Headache characteristics at baseline

Within this cohort 65 participants (98%) reported headache at the time of their IIH diagnosis. At the time of the baseline assessment 63 (95%) reported headache. In some cases patients described more than one type of headache. The headache phenotype was migraine-like in 57 of 63 (90%) and of those, 23 (40%) described migraine aura. Of those with migraine-like headaches 40 (70%) had a phenotype consistent with chronic migraine-like headaches (> 15 headache days per month of which > 8 are migraine-like), while 17 (30%) had episodic migraine-like headaches (less than 15 headache days per month) [8]. At the time of study assessment, 44 (67%) had headache, which fulfilled the diagnostic criteria for headaches attributed to IIH. 23 (35%) patients fulfilled the criteria for medication-overuse headaches. There was one patient who had headaches not attributable to either migraine-like or attributable to IIH, and had tension-type headache.

Headache location was predominately bilateral 76%; with fewer reporting unilateral pain, 20% being on the right side and 18% being on the left side (note these were not mutually exclusive responses, Supplemental Table 1). Headache pain was typically throbbing 73%, but also a pressure sensation 55% and less commonly stabbing 11% or shooting 7%. Photophobia and phonophobia were described in 81% and 60%, respectively, with fewer (19%) describing osmophobia. 70% described nausea and 18% experienced vomiting with their headache attacks. Dizziness was reported in 33%. Headaches on waking were reported in 12%. Autonomic features were rarely reported 5%. Pulsatile tinnitus was a feature in 74%. Headaches were exacerbated by physical activity in 53%; by lying flat in 32%; on bending 31%; and on Valsalva manoeuvre in 23%. (Table 3).

### Headache burden

Headache disability, as measured by the Headache Impact Test (HIT-6) questionnaire, had a mean score of 65 (SD ±7.3) at baseline. Mean headache severity was 5.0 (SD ±2.0) and mean daily duration of headache was 8.2 h (SD ±6.3). Monthly headache days were mean 22.2 days (SD ±7.8) and mean monthly analgesic use was 12.3 days (9.0).

By 12 months 76% of participants reported ongoing headache. The mean headache severity had improved (3.6 (SD ±2.9), with a concurrent reduction in the headache frequency (monthly headache days 15.1 (SD ±11.5)) and monthly analgesic use (8.6 (SD ±9.8)). No parameter reached statistical significance.

### Medications at baseline

At baseline 38% participants were receiving medication specifically to lower ICP. This included 29% receiving Acetazolamide, and 9% Topiramate. 5% of participants were receiving diuretics (Bendroflumethiazide, *n* = 1; Furosemide, *n =* 1; and Co-amilofruse, *n =* 1). 27% of participants were receiving medication for prevention of headache (migraine and/or tension-type headache) (beta blocker, *n =* 1; tricyclic antidepressant, *n* = 7; anticonvulsant, *n* = 8; other, *n* = 2) (Table 2).

**Table 2:** Outcome measures, headache phenotype and ictal cutaneous allodynia at baseline

Headache at baseline, n (%)	63 (95%)
Headache severity, verbal rating scale (0–10), mean (SD)	5.0 (2.0)
Headache duration (hours), mean (SD)	8.2 (6.3)
Headache frequency (days per month), mean (SD)	22.2 (7.8)
Analgesic use (days per month), mean (SD)	12.3 (9.0)
HIT-6 score, mean (SD)	65 (7.3)
SF-36, physical component score, mean (SD) *n =* 60	28.7 (12.7)
SF-36, mental component score, mean (SD) *n =* 60	37.7 (11.0)
Headache phenotypes (not mutually exclusive), n (%)	*n =* 66
No headache	1 (2%)
Migraine-like	57 (86%)
Migraine-like without aura	34 (60%)
Migraine-like with aura	23 (40%)
Chronic migraine-like	40 (70%)
Episodic migraine-like	17 (30%)
Headache attributed to IIH	44 (67%)
Medication-overuse	23 (35%)
Tension-like	1 (2%)
Cutaneous allodynia (ictal), mean (SD), *n* = 58	19.16 (5.79)
None (0–2)	0 (0%)
Mild (3–5)	0 (0%)
Moderate (6–8)	2 (3.4%)
Severe (9+)	56 (96.6%)

### Relationship of ICP to headache

**Fig. 1:**
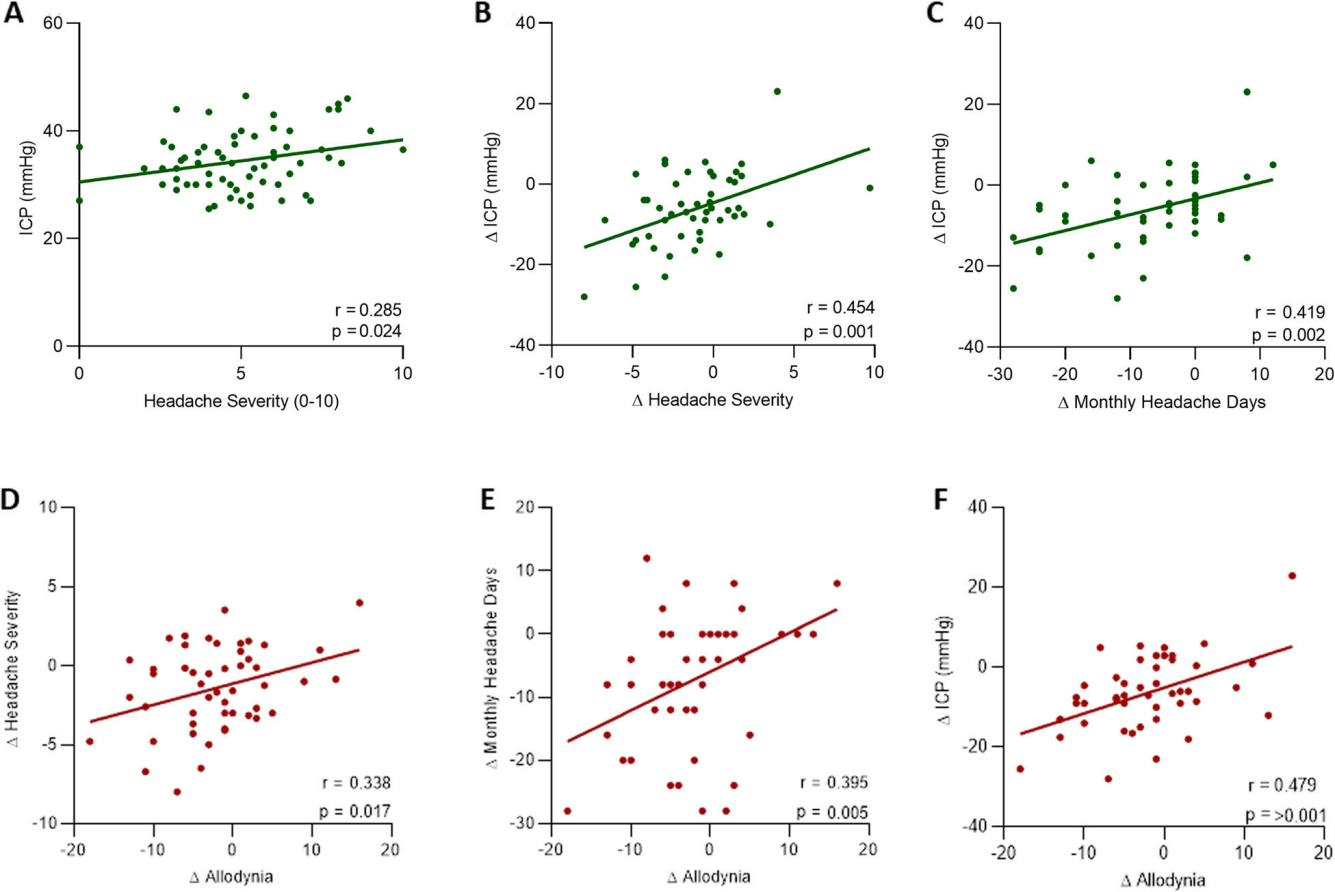
Relationship between ICP and headache. Correlation of baseline headache severity (HS) against baseline intracranial pressure (ICP) (**A**); ICP correlated with change in HS (**B**) change in monthly headache days (MHD) (**C**) Change in MHD correlated with ICP between baseline and 12 months. Correlation of the change allodynia scores between baseline and 12 months against change in HS (**D**); change in MHD (**E**); ICP (**F**) between baseline and 12 months

The whole cohort mean lumbar puncture opening pressure (LP OP) at baseline was 34.7cmCSF (SD 5.7) which reduced to 28.95 (SD 7.7) and 26.8 (SD 8.0) by 12 and 24 months respectively. Headache severity at baseline correlated with ICP (Fig. 1a, *r =* 0.285, *p* = 0.024) although the monthly headache days did not. The reduction in ICP over 12 months correlated with the reduction in headache severity and monthly headache days (MHD) (Fig. 1b and c, *r =* 0.454, *p* = 0.001 and *r =* 0.419, *p* = 0.002 respectively). This relationship continued at 24 months but did not reach statistical significance.

### Relationship between cutaneous allodynia and ICP

At baseline 58/63 (92%) reported ictal cutaneous allodynia with a mean cutaneous allodynia score of 19.2 (SD ± 5.79) and reducing at 12 months to a mean of 17.1 (SD ± 7.20) (*p* = 0.01). There was no relationship between the allodynia score and headache severity and MHD at baseline. By 12 month MHD correlated with the allodynia score (*r =* 0.331, *p* = 0.015). The change in MHD and headache severity over 12 months correlated with the improving allodynia score (Fig. 1d and e, *r =* 0.395, *p* = 0.005 and *r =* 0.338, *p* = 0.017 respectively). The change in allodynia over 12 months also correlated with the change in ICP (Fig. 1e, *r =* 0.479, *p* < 0.001). There was no relationship between body mass index (BMI) and allodynia. Pressure allodynia did not change significantly between baseline and 12 months and was not associated with headache severity, MHD or ICP (Supplemental Table 2).

### Predicting the effects of ICP on headache outcomes

**Fig. 2:**
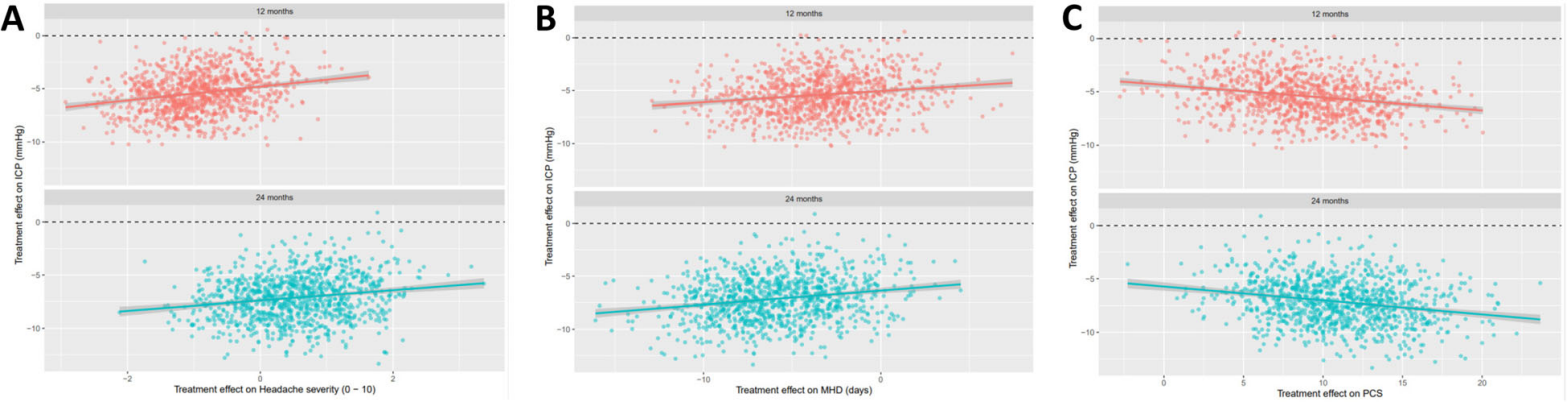
Bootstrap surrogacy analysis of ICP and headache outcomes. The x-axis reflects change in headache outcomes with change in ICP on the y-axis. The lines are simple linear regressions and the shaded regions are 95% confidence intervals of the mean. Changes in intracranial pressure are plotted at 12 and 24 months. Each positive value represent improvement i.e. reduction in ICP and headaches outcomes, with the larger the angle of the slope the greater the relationship. **A** Illustrates headache severity on the y axis with a positive association with ICP over the time horizons. **B** Illustrates monthly headache days (MHD) on the y axis with a positive association with ICP over the time horizons. **C** Illustrates quality of life physical component score (PCS) on the y axis with a positive association with ICP over the time horizons

Analysis identified a positive association between ICP and headache severity and MHD at 12 and 24 months, with a larger change in intracranial pressure coinciding with a larger change in headache measures (Fig. 2a). The bootstrapped analysis data points (Fig. 2) represent trial outcomes, each as likely as any other. Thus the location and dispersion of the points provides evidence on the coincident nature of the change in intracranial pressure. Predictability was observed at all time points (12 and 24 months). Utilizing the surrogacy analysis plots (Fig. 2), changes in headache measures can be inferred from changes in ICP (Table 3) e.g. at 12 months follow up the reduction in ICP of − 5 cmCSF is associated with a change in headache severity of − 0.95 and a mean MHD of − 3.06. Whereas a reduction in ICP of − 10 cmCSF was associated with a reduction of headache severity of − 1.35 and mean MHD of − 4.08, at 12 months (Table 3).

**Table 3:** Predicting changes in headache severity and monthly headache days from changes in intracranial pressure

Time (months)	Change in intracranial pressure (cmH_2_0)	Change in mean headache severity (95% confidence interval)	Change in mean MHD (95% confidence interval)
12	−5	−0.95 (−2.61, 0.68)	−3.06 (−9.61, 2.91)
12	−10	−1.35 (−2.70, 0.10)	−4.08 (−9.56, 2.80)
24	−5	0.43 (−1.18, 1.96)	− 5.32 (− 11.5, 1.35)
24	−10	− 0.19 (− 1.94, 1.61)	−6.17 (− 13.3, 0.79)

### Quality of life and intracranial pressure

**Fig. 3:**
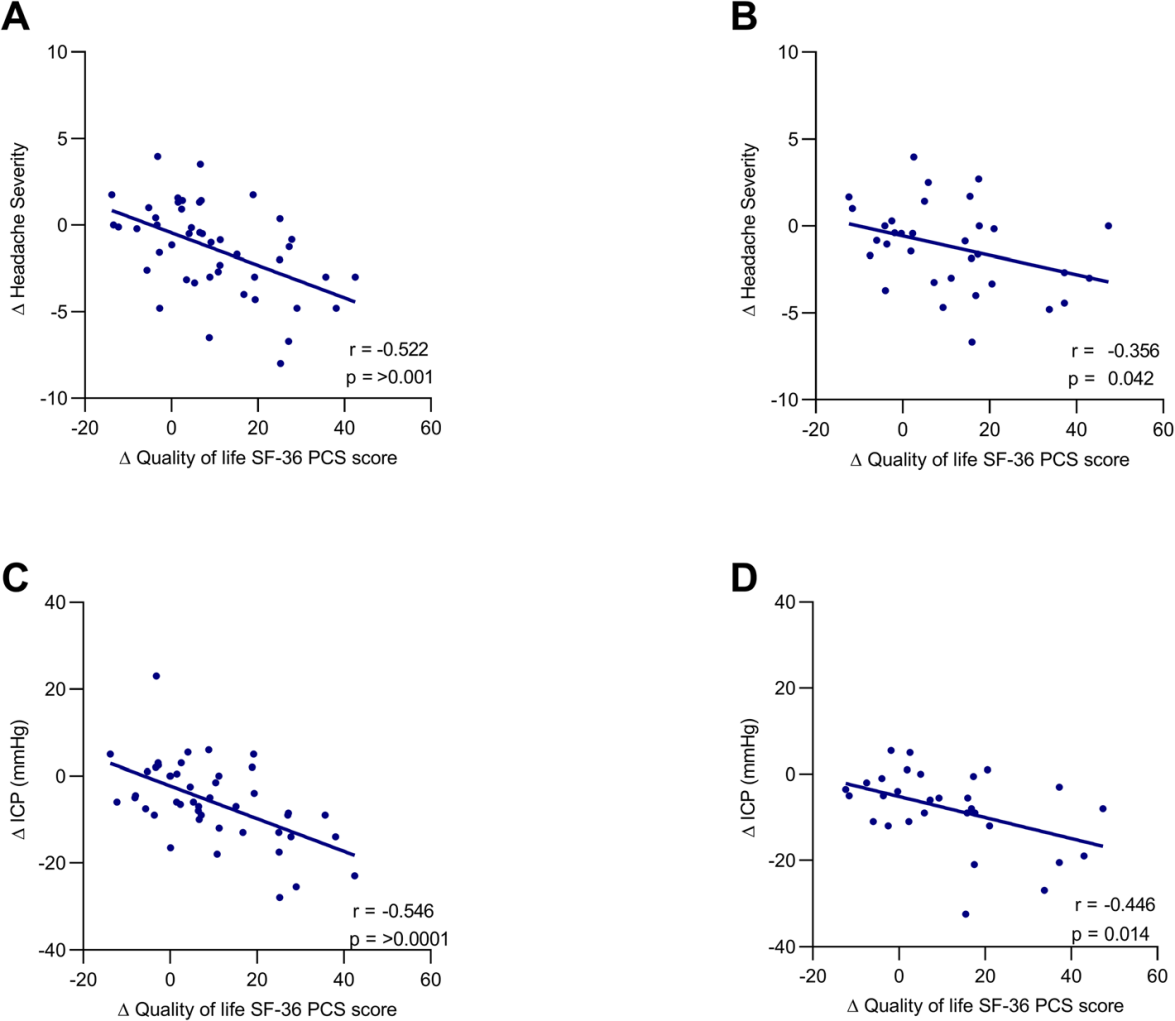
Correlation of change in Quality of Life SF-36 physical component score (PCS) score and change in headache severity (HS) between baseline and 12 months (**A**) baseline and 24 months (**B**); Intracranial Pressure (ICP) between baseline and 12 months (**C**) between baseline and 24 months (**D**)

Quality of life, as measured by SF-36 PCS and MCS were 28.7 (12.7) and 37.7 (11.0) respectively at baseline and 37.7 (14.9) and 38.9 (12.2) respectively by 12 months (Table 2). There was no relationship between SF-36 scores and headache severity, MHD and ICP at baseline. The improvement in the PCS was associated with improving headache severity at 12 and 24 months (Fig. 3a and b, *r =* − 0.522, *p* < 0.001 and *r =* − 0.356, *p* = 0.04, respectively). The PCS also improved in association with reduction in ICP at 12 months (*r =* − 0.546, *p <* 0.001). MCS did not relate to headache or ICP. Analysis identified a positive association between ICP and PCS at 12 and 24 months (Fig. 3C).

Subanalysis by treatment assignment and disease duration was not possible due to small numbers in each group.

## Discussion

Headache is a near universal sequela of IIH, and can complicate other disorders with raised ICP. We demonstrate a positive relationship between ICP and headache severity and monthly headache days, which has not been noted previously in IIH. Patients with the greatest reduction in ICP over 12 months saw the greatest reduction in headache frequency and severity, and this was associated with improvement of physical functioning in the quality of life SF-36.

We observed that the majority of patients with IIH had migraine-like headaches at baseline, as previously reported [15, 18]. One patient was headache-free, whereas the majority described severe continuous daily headache pain associated with poor quality of life. Overall headache in patients with active IIH was of moderate pain severity, long daily duration and a mean frequency of 22 days per month, with the associated headache impacting on the individual’s quality of life. These observations are in keeping with broader patients’ views that the chronic daily headache of IIH is very disabling [6] and is already known to drive reduction in quality of life in IIH [8].

A previous randomized controlled trial in IIH [15] classified 68% with migraine or probable migraine and 26% tension-type or probable tension-type headaches. In the present study 90% reported migraine-like headaches and 70% would fulfil the criteria for a diagnosis of chronic migraine. Furthermore, 40% of participants in the present study described aura. In our cohort tension-type headaches were not common, with only one patient fulfilling the IHS criteria [9]. This may reflect differences between the types of patients recruited to the two trials, for example the IIHTT [15] participants were newly diagnosed patients, whereas the IIH:WT participants reflected a more chronic disease duration (Table 1).

68% of participants reported a prior diagnosis of migraine before being diagnosed with IIH, of whom over half (53%) had been diagnosed with migraine prior to the age of 18 years old and only 11% developed migraine-like headaches following the diagnosis of IIH. This portion of patients with a prior migraine history is considerably higher than that of the general population, where for example one study found the lifetime prevalence of migraine to be 29% [19]. A number of interesting observations could be postulated, for example, those with a recent diagnosis of migraine could have been initially diagnosed as migraine instead of IIH, however the portion of those with a very longstanding history of migraine are unlikely to have been misdiagnosed. Given the longevity of migraine in our cohort, some patients may have had chronic migraine before diagnosis of IIH, whereas others may have developed chronic migraine-like headaches following diagnosis. It remains unclear whether diagnosis of IIH in patients with known episodic migraine contributes towards transition to chronic migraine and if this is dependent on central sensitization.

Cutaneous allodynia is reported in over half of all patients with migraine [20]. It has not typically been a feature of conditions associated with raised ICP (e.g. brain tumours or hydrocephalus). A previous study reported allodynia in 50% of IIH patients who mostly had a migraine-like headache profile [18]. In the present study 92% reported cutaneous allodynia at the maximum headache severity and although this was not associated with headache severity or frequency at baseline, change in allodynia over 12 months was associated with change in ICP.

Headache management is an unmet need in this disease, with no randomised controlled trials to guide treatment options. Only 18 participants were on concurrent headache preventative therapies, whereas 40 fulfilled the criteria for chronic migraine-like headaches, leaving a large portion of patients under treated. Recently the first prospective open label study of a CGRP monoclonal receptor antibody reported substantial improvements at 3 months, which continued for up to 12 months in the reduction of monthly moderate/severe headache days [13]. This benefit was seen both in those with and without prior migraine and in those with or without prior existing medication overuse headache [13]. Topiramate, a well-known migraine preventative therapy has been evaluated in IIH in the context of the impact on vision, rather than its beneficial effects of reduction of headache disability and is used off label in routine clinical practice [21]. However consensus guidelines for IIH placed topiramate as a useful medication for management of headaches in IIH in order to avoid medicines such as beta-blockers and tricyclics that may exacerbate weight gain, a known precipitant of the disease [1].

Similar to a previous study approximately a third of participants met the diagnostic criteria for medication-overuse headache at baseline [9]. Medication-overuse headache is particularly common in patients with a background of chronic migraine, for example in the United States it has been reported in up to a quarter. However, there are many factors that influence medication-overuse headache [22, 23]. Advising patients about appropriate usage of headache analgesics and avoidance of opiates is therefore an important part of headache management in IIH [1].

The data presented indicates a relationship between increased ICP and increased presence of migraine-type headache. Change in ICP can predict change in headache over time. In a previous randomized controlled trial of those with recent onset IIH (within 2 weeks of presentation) no correlation between headache characteristics and ICP was found over a 6 month follow-up [15]. Future studies are required to investigate this complex relationship from the acutely presenting patient to the more chronic phase of IIH headache. ICP monitoring studies may further delineate this complex relationship further. Therapies that have been shown to reduce ICP in animal models, such as GLP-1 [24], could be helpful in both reducing headache burden in conditions of raised ICP and weight management in IIH [25].

## Conclusions

This detailed prospective evaluation of headache in patients with IIH demonstrates a consistent relationship between headache (severity and monthly headache days) and ICP. Modelling of this relationship between headache and ICP enabled prediction of headache outcomes depending on changes in ICP over a 12 and 24 month horizon. The headache in patients with active IIH is migraine-like in the majority. IIH patients demonstrated markers of allodynia which improved with reduction in ICP. Quality of life measures improved with reduction in ICP and headache. Therapeutic strategies to improve ICP are likely to improve headache in IIH.

## Supplementary Information

**Additional file 1: Supplemental methods** - allodynia checklist.

**Additional file 2: Supplemental Table 1-** Headache characteristics at baseline.

**Additional file 3: Supplemental Table 2 –** Pressure allodynia at baseline and 12 months.

## Data Availability

Data will be made for reasonable requests.

## Abbreviations

ASC-12: Allodynia Symptom Checklist-12
CGRP: Calcitonin gene-related peptide
CSF: Cerebrospinal fluid
HS: Headache severity
ICP: Intracranial pressure
IIH: Idiopathic intracranial hypertension
IIH:WT: IIH weight trial
IHS: International Headache Society
LP: Lumbar puncture
MHD: Monthly headache days
NRS: Numerical rating system
OP: Opening pressure
PCS: Physical component score
