# Supplementary material for: Intracranial pressure directly predicts headache morbidity in idiopathic intracranial hypertension
Source: J Headache Pain. 2021 Oct 7;22(1):118. doi: 10.1186/s10194-021-01321-8 (PMC8499560; doi:10.1186/s10194-021-01321-8)
Supplement: Supplementary file 1 — Additional file 1: Supplemental methods - allodynia checklist. [file 10194_2021_1321_MOESM1_ESM.docx]

**Supplemental methods** - allodynia checklist:

**Allodynia symptom checklist-12**

The allodynia symptom checklist response scales are coded as follows:

- Combing your hair: Does not apply=0, Never=0, Rarely=0, Less than half the time=1, Half the time or more=2
- Pulling your hair back: Does not apply=0, Never=0, Rarely=0, Less than half the time=1, Half the time or more=2
- Shaving your face: Does not apply=0, Never=0, Rarely=0, Less than half the time=1, Half the time or more=2
- Wearing eyeglasses: Does not apply=0, Never=0, Rarely=0, Less than half the time=1, Half the time or more=2
- Wearing contact lenses: Does not apply=0, Never=0, Rarely=0, Less than half the time=1, Half the time or more=2
- Wearing earrings: Does not apply=0, Never=0, Rarely=0, Less than half the time=1, Half the time or more=2
- Wearing necklace: Does not apply=0, Never=0, Rarely=0, Less than half the time=1, Half the time or more=2
- Wearing tight clothing: Does not apply=0, Never=0, Rarely=0, Less than half the time=1, Half the time or more=2
- Taking a shower: Does not apply=0, Never=0, Rarely=0, Less than half the time=1, Half the time or more=2
- Resting your face or head on a pillow: Does not apply=0, Never=0, Rarely=0, Less than half the time=1, Half the time or more=2
- Exposure to heat: Does not apply=0, Never=0, Rarely=0, Less than half the time=1, Half the time or more=2
- Exposure to cold: Does not apply=0, Never=0, Rarely=0, Less than half the time=1, Half the time or more=2

The total score is calculated by summing the values for each question.

The score ranges from 0 to 24 where a low score is good

The allodynia scores can also be characterised using cut-off values as follows:

0-2 = no allodynia

3-5 = mild allodynia

6-8 = moderate allodynia

9 or more = severe allodynia
