# Supplementary material for: Intracranial pressure directly predicts headache morbidity in idiopathic intracranial hypertension
Source: J Headache Pain. 2021 Oct 7;22(1):118. doi: 10.1186/s10194-021-01321-8 (PMC8499560; doi:10.1186/s10194-021-01321-8)
Supplement: Supplementary file 2 — Additional file 2: Supplemental Table 1- Headache characteristics at baseline. [file 10194_2021_1321_MOESM2_ESM.docx]

**Supplemental Table 1-** Headache characteristics at baseline

|  |  |
| --- | --- |
| Headache location (not mutually exclusive), % (number) |  |
| Bilateral | 76% (37/49) |
| Unilateral Right | 20% (10/49) |
| Unilateral Left | 18% (9/49) |
| Frontal | 72% (31/43) |
| Temporal | 47% (20/43) |
| Parietal | 37% (16/43) |
| Occipital | 44% (19/43) |
| Periorbital | 8% (3/38) |
| Retroorbital | 18% (7/38) |
| Headache quality (not mutually exclusive), % (number) |  |
| Throbbing | 73% (32/44) |
| Pressure | 55% (24/44) |
| Stabbing/Sharp | 11% (5/44) |
| Shooting | 7% (3/44) |
| Unclear | 35% (17/58) |
| Combination of features | 45% (20/44) |
| Associated symptoms, % (number) |  |
| Photophobia | 81% (38/47) |
| Phonophobia | 60% (27/45) |
| Osmophobia | 19% (3/16) |
| Nausea | 70% (32/46) |
| Vomiting | 18% (8/45) |
| Dizzy symptoms | 33% (10/30) |
| Autonomic features | 5% (1/19) |
| Pulsatile tinnitus | 74% (49/66) |
| Activity that exacerbated headache |  |
| Physical activity | 53% (19/36) |
| Lying flat | 32% (9/28) |
| Bending | 31% (11/35) |
| Valsalva manoeuvre | 23% (8/35) |
