# Supplementary material for: Intracranial pressure directly predicts headache morbidity in idiopathic intracranial hypertension
Source: J Headache Pain. 2021 Oct 7;22(1):118. doi: 10.1186/s10194-021-01321-8 (PMC8499560; doi:10.1186/s10194-021-01321-8)
Supplement: Supplementary file 3 — Additional file 3: Supplemental Table 2 – Pressure allodynia at baseline and 12 months. [file 10194_2021_1321_MOESM3_ESM.docx]

**Supplemental Table 2 –** Pressure allodynia at baseline and 12 months

|  | Baseline | | |  | 12 months | | |
| --- | --- | --- | --- | --- | --- | --- | --- |
|  | n | mean | sd |  | n | mean | sd |
|  |  |  |  |  |  |  |  |
| F1 | 54 | 0.54 | 1.00 |  | 45 | 0.34 | 0.77 |
| F2 | 54 | 2.10 | 2.53 |  | 45 | 1.52 | 2.60 |
| F3 | 54 | 7.42 | 8.49 |  | 45 | 7.69 | 11.47 |
| HS (0-10) | 59 | 5.02 | 2.00 |  | 49 | 3.66 | 2.88 |
| MHD / days | 59 | 22.31 | 7.76 |  | 49 | 15.67 | 11.48 |
| ICP / cmCSF | 62 | 34.68 | 5.69 |  | 51 | 28.88 | 7.84 |

Key: sd, standard deviation; von Frey hairs (F1, 0.32g; F2, 8.30g; and F3, 24g); HS, headache severity; MHD, monthly headache days; ICP, intracranial pressure.
